# Human Febrile Illness Caused by Encephalomyocarditis Virus Infection, Peru

**DOI:** 10.3201/eid1504.081428

**Published:** 2009-04

**Authors:** M. Steven Oberste, Eduardo Gotuzzo, Patrick Blair, W. Allan Nix, Thomas G. Ksiazek, James A. Comer, Pierre Rollin, Cynthia S. Goldsmith, James Olson, Tadeusz J. Kochel

**Affiliations:** Centers for Disease Control and Prevention, Atlanta, Georgia, USA (M.S. Oberste, W.A. Nix, T.G. Ksiazek, J.A. Comer, P. Rollin, C.S. Goldsmith); Universidad Peruana Cayetano Heredia, Lima, Peru (E. Gotuzzo); Naval Medical Research Center Detachment, Lima (P. Blair, J. Olson, T.J. Kochel)

**Keywords:** Picornavirus, cardiovirus, encephalomyocarditis virus, febrile illness, human infections, Peru, research

## Abstract

Etiologic studies of acute febrile disease were conducted in sites across South America, including Cusco and Iquitos, Peru. Patients’ clinical signs and symptoms were recorded, and acute- and convalescent-phase serum samples were obtained for serologic examination and virus isolation in Vero E6 and C6/36 cells. Virus isolated in Vero E6 cells was identified as encephalomyocarditis virus (EMCV) by electron microscopy and by subsequent molecular diagnostic testing of samples from 2 febrile patients with nausea, headache, and dyspnea. The virus was recovered from acute-phase serum samples from both case-patients and identified with cardiovirus-specific reverse transcription–PCR and sequencing. Serum samples from case-patient 1 showed cardiovirus antibody by immunoglobulin M ELISA (acute phase <8, convalescent phase >1,024) and by neutralization assay (acute phase <10, convalescent phase >1,280). Serum samples from case-patient 2 did not contain antibodies detectable by either assay. Detection of virus in serum strongly supports a role for EMCV in human infection and febrile illness.

## Abstract 2

Encephalomyocarditis virus was identified in the serum of 2 febrile patients in Peru.

Encephalomyocarditis virus (EMCV; family *Picornaviridae*, genus *Cardiovirus*) is a group of closely related virus strains belonging to 1 serotype with a wide host range (*1*). Infection with EMCV is associated with sporadic cases and outbreaks of myocarditis and encephalitis in domestic pigs, in numerous species of nonhuman primates, and in other mammalian species (*2**–**8*). The disease is often fatal—frequently, sudden death is the first indication of infection—and most outbreaks have been associated with captive animals, such as those found in piggeries, primate research centers, and zoos. Virus isolation and serologic studies indicate EMCV is distributed worldwide, but clinical disease in humans or domestic animals is relatively infrequent. Although disease transmission is poorly understood, rodents appear to be the natural reservoirs (*1*). Rodent infestation has been implicated in the genesis of several epizootics; disease transmission apparently results from close contact between rodents or their excreta and individuals of susceptible mammalian species (*1**,**2**,**4**,**5**,**8**,**9*). In several instances, rodent control measures have interrupted disease transmission and halted institutional epizootics (*4*).

Human EMCV infection and disease have been documented by virus isolation from several specimen types, including serum, stool samples, cerebral spinal fluid, and throat washings (*10**–**12*). However, because this disease is so infrequent in humans, positive association with EMCV is difficult to establish. In addition, results of several early studies were questionable because EMCV was isolated by using laboratory mice; researchers could not unequivocally establish that the virus did not originate from the mice used for isolation and passage rather than from human clinical specimens (*12*). Recently, a novel cardiovirus, Saffold virus (SAFV; genus *Theilovirus*), was reported in association with fever of unknown origin in a child 8 months of age (*13*) and in children with symptoms of respiratory or gastrointestinal illness (*14**–**16*). This finding suggests that additional cardioviruses may be pathogenic for humans. Serologic studies also indicate that humans have been infected by EMCV or immunologically related viruses (*17**–**22*). Antibody prevalence varied somewhat from study to study, but seropositivity rates tended to increase in persons of advancing age, consistent with a continuous risk for infection throughout life. In early 2004, we identified EMCV infection in serum samples from 2 febrile patients in Peru.

## Materials and Methods

### Surveillance System

In collaboration with the Peruvian Ministry of Health, the Naval Medical Research Center Detachment, Lima, Peru, has established a febrile illness surveillance program designed to identify the causes of febrile diseases in the country. The program carries out passive surveillance in Ministry of Health posts, where febrile patients are identified, signs and symptoms are recorded, and acute- and convalescent-phase blood samples are collected for diagnostic studies. Tests routinely performed in support of the surveillance program include immunoglobulin (Ig) M ELISA for flaviviruses, alphaviruses, bunyaviruses, arenaviruses, and rickettsia, as well as virus isolation, reverse transcription–PCR (RT-PCR), and sequencing. The study protocol was approved by the Naval Medical Research Center Institutional Review Board (Protocol NMRCD.2000.0006, Department of Defense 31535), in compliance with all federal regulations governing the protection of human patients; the protocol also was reviewed by the Peruvian Ministry of Health.

### Case Definitions

Clinical diagnoses were considered confirmed if laboratory testing resulted in isolation of virus from the specimen, virus detection by RT-PCR, or a 4-fold or greater increase in IgM antibody titers. Diagnoses were considered presumptive if the IgM titer was elevated in the acute-phase sample only or a 4-fold or greater increase was discerned upon comparison of acute- and convalescent-phase titers. In the absence of laboratory evidence of a specific pathogen, cases were classified as negative.

### Virus Isolation

Viruses were isolated by using a modification of a published protocol (*23*). Acute-phase serum samples, obtained no more than 5 days after disease onset, were transported on dry ice to the Naval Medical Research Center Detached laboratory in Lima and stored at –80°C. Serum samples were thawed and diluted 1:5 in minimum essential medium containing 2% heat-inactivated fetal bovine serum and antimicrobial agents. African green monkey (Vero) (37°C) and/or mosquito C6/36 (28°C) cell cultures were each injected with 200 μL of the diluted serum into 25-cm^2^ flasks. Upon observation of viral cytopathic effect, or 10 days postinoculation (dpi) if no cytopathic effect was observed, cells were removed from the flasks and placed on 12-well glass spot-slides for examination by immunofluorescence assay using group- and virus-specific polyclonal and monoclonal antibodies. The antibodies used were reactive with dengue viruses 1–4, yellow fever virus, St. Louis encephalitis virus, Rocio virus, Ilheus virus, West Nile virus, Venezuelan equine encephalitis virus, eastern equine encephalitis virus, Mayaro virus, Trocara virus, Oropouche virus, Caraparu virus, Murtucu virus, Guaroa virus, hantavirus, Machupo virus, and Tacaribe virus; after the cardiovirus isolation, EMCV antibody also was used.

Upon receipt of the viruses at the Centers for Disease Control and Prevention (CDC; Atlanta, Georgia, USA), a master seed stock was prepared from each virus and working stocks were made by passage in Vero E6 cells. Cytopathic effect developed rapidly, and the cultures were harvested at 3–4 dpi; some infected cells were fixed in 2.5% glutaraldehyde for electron microscopy examination. For virus reisolation at CDC, acute-phase serum samples were injected into Vero E6 cells. Virus isolates were obtained in Vero E6 cells from acute-phase serum of both case-patients. The isolates, IQD6726 (Iquitos) and FSC575 (Cusco), were passaged in Vero E6 cells to obtain stocks for further analysis. Litters of suckling mice were inoculated intracerebrally with both strains of virus. Brain, spleen, and liver tissues were harvested and fixed in 2.5% glutaraldehyde for electron microscopic examination.

### RNA Extraction, RT-PCR, and Sequencing

RNA was extracted from the virus isolates by using the QIAamp Viral RNA Mini Kit (QIAGEN, Valencia, CA, USA). cDNA reactions consisted of 2 μL of extracted viral RNA, 50 ng of random hexamers (Applied Biosystems, Foster City, CA, USA), 4 μL of 5× first strand buffer (Invitrogen, Carlsbad, CA, USA), 200 μmol/L of each dNTP (GE Healthcare, Piscataway, NJ, USA), 40 U of RNasin ribonuclease inhibitor (Promega, Madison, WI, USA), and 200 U of SuperScript II reverse transcriptase (Invitrogen) in a total volume of 20 μL. The cDNA reactions were incubated for 10 min at 25°C, followed by 45 min at 42°C and 4 min at 95°C. For the PCR assays, 3 genome regions were targeted, the 5′-NTR, VP1, and 3D regions (Table). Two sets of PCR primers targeting the viral protein 1 (VP1) gene were used, yielding overlapping PCR fragments. PCR reactions consisted of 2 μL of cDNA, 200 μmol/L of each dNTP (GE Healthcare), 5 μL of 10× buffer with MgCl_2_ (Roche Molecular Biochemicals, Indianapolis, IN, USA), 2.5 U of Fast Start Taq DNA Polymerase (Roche), and forward and reverse primers (10 pmol of each of AN312 and AN315; 40 pmol each of AN283, AN285, AN393, and AN286; 20 pmol each of P1 and P2; and 60 pmol each of 1C340F and 2B188R), in a total volume of 50 μL. Cycling parameters were 42ºC for 50 min, 50°C for 10 min, and 95ºC for 5 min, followed by 45 cycles of 95ºC for 30 sec, 45ºC for 40 sec, and 60ºC for 1 min. All PCR products were analyzed by electrophoresis in 2% agarose gels stained with ethidium bromide.

**Table:** Oligonucleotide primers used for cardiovirus PCR amplification and sequencing*

Primer	Sequence (5′ → 3′)	Region	Coordinates	Reference
AN312	GARTVWCGYRAAGRAAGCAGT	5′-NTR	455–475	This study
AN315	GGYRCTGGGGTTGYRCCGC	5′-NTR	618–600	This study
AN283	GCAGACGGWTGGGTNACNGTNTGG	VP3	2559–2582	This study
AN285	AGAGTAACCTCTACRTCRCAYTTRTA	VP1	3097–3072	This study
AN393	TTTCCACTCAAGTCTAARCARGAYT	VP1	3015–3049	This study
AN286	AAGAAGACAGTCGGACGNGGRCARAANAC	VP1	3472–3444	This study
P1	CCCTACCTCACGGAATGGGGCAAAG	3D	7655–7631	(*24*)
P2	GGTGAGAGCAAGCCTCGCAAAGACAG	3D	7370–7395	(*24*)

*NTR, nontranslated region; VP1, viral protein 1.

Amplicons were purified from an agarose gel (QIAGEN gel extraction kit) before sequencing. Both strands were sequenced by using the PCR primers, a Prism BigDye Terminator version 1.1 or 3.1 Ready Reaction Cycle Sequencing kit (Applied Biosystems), and a model 3100 or 3130 Genetic Analyzer (Applied Biosystems). The sequences determined in this study were deposited in the GenBank sequence database, accession nos. EU979543–EU979548.

Sequences were identified in GenBank by using BLAST to compare sequences (www.ncbi.nlm.nih.gov/blast). For phylogenetic reconstruction, sequences were aligned with those of other cardioviruses by using PileUp (Wisconsin Sequence Package, version 11.1; Accelrys, San Diego, CA, USA), and trees were generated with the neighbor-joining method implemented in ClustalX version 1.83 (*25*). Genetic distances were estimated by the Kimura 2-parameter method. To assess the confidence of branching patterns of the neighbor-joining tree, 1,000 bootstrap replicates were performed.

### Serologic Testing

Serum specimens were tested for neutralizing antibody against EMCV by a modified microneutralization assay (*26*). Approximately 80–100 median 50% cell culture infectious dose (CCID_50_) of isolate FSC575 and serial dilutions of serum (starting at 1:10 and ending at 1:1,280) were incubated together at 37°C for 2.5 h before Vero E6 cells were added to the wells. After incubation for 5 days at 37°C, each plate was stained with 0.05% crystal violet in 25% ethyl alcohol and, after drying, the optical density in each well was measured at 570 nm. Each specimen was run in triplicate; the final titer was estimated by use of the Spearman-Karber method (*27*). Positive-control anti-EMCV serum was generously provided by Ann Palmenberg.

Pathogen-specific IgM titers were determined by using an adapted IgM-capture ELISA (*28*). Briefly, 96-well plates were coated with antihuman IgM antibody to capture patient IgM molecules. Virus-specific IgM was detected by adding viral antigen, followed by virus-specific mouse hyperimmune ascitic fluid and horseradish peroxidase-labeled antimouse IgG. After the addition of colorimetric substrate, absorbance was read at 490 nm. All acute- and convalescent-phase samples were initially screened at 1:100. Samples exceeding the reference cutoff value, calculated as the median of 7 antibody-negative samples plus 3 standard deviations, were considered IgM positive. Positive samples were subsequently retested by using 4-fold serial dilutions to determine end-point titers. Viruses included in the ELISAs included dengue viruses 1–4, yellow fever virus, St. Louis encephalitis virus, Rocio virus, Ilheus virus, West Nile virus, Venezuelan equine encephalitis virus, eastern equine encephalitis virus, Mayaro virus, Trocara virus, Oropouche virus, Caraparu virus, Murtucu virus, Guaroa virus, hantavirus, Machupo virus, and Tacaribe virus; after the cardiovirus isolation, EMCV was also used.

## Results

### Clinical Description of Cases

The first patient was identified in the Amazonian city of Iquitos, Loreto Department, Peru. Iquitos (3°44.69′S, 73°15.25′W) is situated at an elevation of 120 m and has a population of ≈350,000. The city is surrounded by the Nanay, Itaya, and Amazon rivers and is accessible only by air or river. The climate is tropical, mean temperature is 25°C, and annual rainfall is 2.7 m. Rodents are common in urban and rural areas; swine and primates are abundant in rural zones.

Case-patient 1 was a 59-year-old housewife from Iquitos. She sought treatment at Hospital Apoyo Iquitos after 3 days of fever, pallor, poor appetite, malaise, nausea, and headache. She recovered completely from fever after 6 days of treatment as an outpatient. Acute-phase (3 days postonset), convalescent-phase (15 days postonset), and late convalescent–phase (8 months later) blood samples were obtained. She had no identifiable focus of infection, negative thick and thin smears for malaria, and a negative tourniquet test. She reported no yellow fever or hepatitis B vaccination and no travel outside of Iquitos within 2 weeks before onset. The patient was interviewed 8 months after seeking treatment when virus isolation results were completed. She reported only limited contact with rats and cats in her house and a neighbor’s house where she played bingo frequently. After 7 days and nights of trapping in both houses and in a sewer adjacent to the neighbor’s house, no rodents were collected.

The second case-patient was identified in the Cusco Regional Hospital but had traveled from Quebrada, a small rural village on the Yanatile River, about 8 hours travel time from the city of Cusco. Quebrada (approximately 13°55′S, 71°40′W) is located in Yanatile district, Calca Province, in the Department of Cusco. The weather in Quebrada is warmer than that in Cusco (temperate climate) at an altitude of 2,926 m. Malaria and leishmaniasis are endemic to the area. The city is accessible by air and land from Cusco.

Case-patient 2 was a 39-year-old male farmer from Cusco-Acomayo/Quebrada Calca. He sought treatment at the local hospital after 7 days of fever. Additional symptoms were headache, malaise, retro-ocular pain, sweats, weight loss, arthralgia, photophobia, poor appetite, myalgia, chills, pallor, nausea, vomiting, and abdominal pain. He was hospitalized with a diagnosis of febrile syndrome and urinary tract infection (UTI). Urinalysis showed 20–25 leukocytes per high-power field. The patient had elevated values for alkaline phosphatase (680 U/L; reference range 68–240 U/L), total bilirubin (1.68 mg/dL; reference value <1.0 mg/dL), and direct bilirubin (1.06 mg/dL; reference value <0.2 mg/dL). Thick and thin smears for malaria and *Bartonella* spp. were negative, as were urine culture and a tourniquet test for dengue hemorrhagic fever. The patient was treated with oral ciprofloxacin (750 mg 2×/d for 7 days), oral paracetamol (acetaminophen; 500 mg as needed for fever >38.5ºC), and intravenous fluids. He recovered completely after 7 days as an inpatient. At the time of his convalescent-phase sample (15 days postonset), his physical examination was within normal limits. He reported tuberculosis in a family member contact (treated for 5 months), yellow fever vaccination in 1994, and no vaccination against hepatitis B. No case investigation was carried out for this patient to determine risk factors for disease. However, the typically rural populations near Cusco have contact with a variety of animal species such as mules, dogs, cats, swine, rodents, rabbits, llamas, alpacas, and vicuñas.

### Virus Isolation and Identification

**Figure 1:**
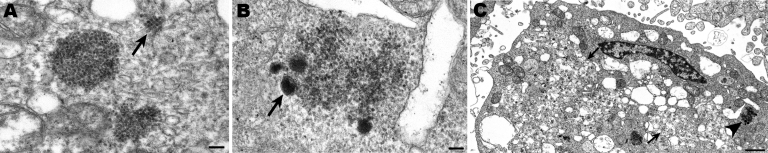
Ultrastructural morphologic features of cardiovirus-infected Vero E6 cells. A) Collections of picornavirus particles, some arranged in a paracrystalline array (arrow). Scale bar = 100 nm. B) Higher magnification of area pointed to by arrowhead in panel C showing condensed material (arrow) at periphery of a viral cluster. Scale bar = 100 nm. C) Cardiovirus-infected cell, showing membrane proliferation and vesiculation (arrows). Scale bar = 1 μm.

Virus isolates were obtained from acute-phase serum specimens of both patients by inoculation of Vero E6 cell cultures. Electron microscopic studies of infected Vero E6 cells and mouse tissues demonstrated cytoplasmic accumulations of particles consistent with the features of picornaviruses. Virions averaged 24 nm in diameter and were occasionally found in paracrystalline arrays (Figure 1, panel A). The infected cells were notable for areas of vesiculation and membrane proliferation (Figure 1, panel C), consistent with the replication complexes, which have been described for picornavirus-infected cells (*29*). Newborn mice inoculated intracerebrally all died at 3 dpi. Rapid death in neonatal mice, coupled with ultrastructural evidence of picornavirus infection, was consistent with the presence of a cardiovirus.

**Figure 2:**
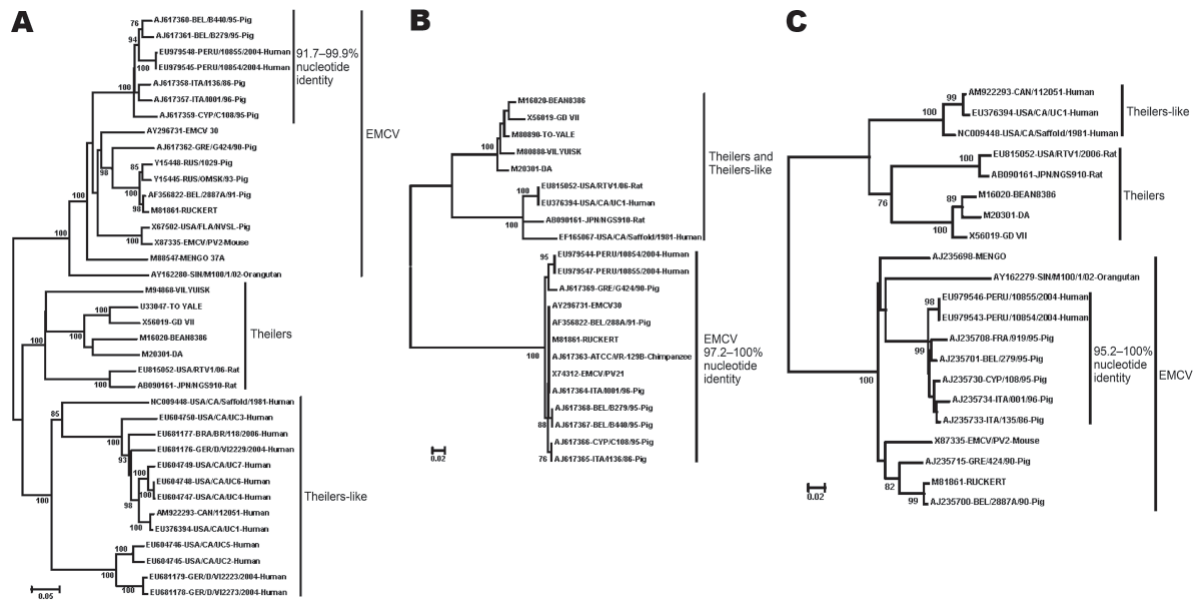
Phylogenetic relationships among viruses detected in Peru and other encephalomyocarditis viruses (EMCVs), and their relationship to the Theiler and Theiler-like cardioviruses. A) Viral protein 1 (VP1); 737 nucleotides (90% of the VP1 gene). The missing portion of the VP1 gene is at the 3’ end. B) 5′ nontranslated region; 145 nucleotides consisting of a highly conserved portion of the internal ribosome entry site, sequence coordinates 558 to 699 relative to EMCV GenBank accession no. AX786477. C) 3D; 210 nucleotides (15% of the 3D gene). The use of this portion of the 3D gene for phylogenetic analysis has been described elsewhere (*25*). Phylogenies were reconstructed with the neighbor-joining method implemented in ClustalX version 1.83 (*27*). Genetic distances were estimated by the Kimura 2-parameter method. To assess the confidence of branching patterns, 1,000 bootstrap replicates were performed. Cardioviruses are identified by GenBank accession number and strain name; when available, complete virus information is given, using the following convention: GenBank accession number, country of origin/strain name/year of isolation-host species. Country abbreviations: BEL, Belgium; BRA, Brazil; CAN, Canada; CYP, Cyprus; FRA, France; GER, Germany; GRE, Greece; ITA, Italy; JPN, Japan; RUS, Russia; SIN, Singapore; USA, United States. Scale bars indicate number of nucleotide substitutions per site.

To confirm the diagnosis, we used cardiovirus-specific RT-PCR assays targeting the 5′-nontranslated region and the VP1 and 3D coding regions (Table). Each of the cardiovirus-specific primer sets amplified a specific DNA product of the expected size; in each instance, the identity of the amplicon was confirmed by sequencing. In a phylogenetic reconstruction done on the basis of sequences from the VP1 capsid region, the Peru viruses clustered with sequences of EMCV strains derived from pigs from Belgium, Cyprus, and Italy; the EMCV reference strains and field strains from pigs and orangutans formed separate subgroups within the EMCV species group (Figure 2, panel A). The VP1 nucleotide sequences of the Peru strains were 91%–100% identical to those of other strains within their phylogenetic subgroup. VP1 nucleotide identities of the Peru cardioviruses to the other EMCV subgroups ranged from 71% to 80% and from 47% to 51% to the rodent and human viruses in the *Theilovirus* genus, respectively. The Peru strains were also most closely related to the European pig strains in the 5′-NTR and 3D regions (Figure 2, panels B, C). Within the larger EMCV 5′-NTR group, containing the Peru cardioviruses, pairwise nucleotide identities ranged from 95.8% to 100.0% (Figure 2, panel B). Relatedness of the Peru cardioviruses’ 5′-NTR to the EMCV and Theiler’s viruses outside of this group ranged from 30.3% to 58.4%. Within the large 3D clade containing the Peru cardioviruses and European pig strains (Figure 2, panel C), the nucleotide identities ranged from 95.2% to 100.0%. 3D nucleotide identities of the Peru cardioviruses to the EMCVs outside the Peru clade ranged from 80.0% to 87.6%. Relatedness of the Peru cardioviruses’ 3D region to the Theiler’s viruses ranged from 56.7% to 61.4%.

### Serologic Confirmation of Infection

Serum from case-patient 1 was seropositive for cardiovirus by IgM monoclonal antibody-capture ELISA; titer was high. Seroconversion was documented by an increase in titer from <8 in the acute-phase sample (day 3) to >1,024 in the convalescent-phase sample (day 15). The neutralization assay yielded similar results; acute-phase titer was <10 and a convalescent-phase titer was >1,280. Serum samples from case-patient 2, collected on days 7 and 15, were negative in both assays (titers <8 and <10, respectively), despite the presence of virus in the day 7 serum sample.

## Discussion

Our ongoing febrile surveillance studies identified and documented 2 cases of human EMCV disease, each of which was diagnosed by virus isolation from acute-phase serum. One patient had an undifferentiated fever with complete recovery after 6 days of fever. The second patient’s disease was complicated by concomitant UTI, which presumably prolonged the course of disease (11 days of fever). Even though EMCV was isolated from this patient, we cannot be certain whether specific symptoms resulted from the viral infection or from the concurrent UTI. At the time of hospitalization, the urine culture was negative, but the patient had received a course of antimicrobial drugs before his hospitalization. However, the urinary tract symptoms also could be consistent with EMCV infection because orchitis has been observed in male laboratory male mice and hamsters (*30**,**31*). Hyperbilirubinemia, associated with EMCV infection in humans, is consistent with the observation of liver compromise that has been well documented in laboratory studies of EMCV-infected mice, swine, and primates (*5**,**32*). The 2 case-patients probably had contact with infected animals. The proposed routes of transmission to humans are contamination of wounds and contact with domestic animals, such as cats, that frequently contact EMCV-infected rodents. The virus identified was distinct from EMCV reference strains but closely related to EMCV strains from pigs in Europe in all 3 genomic regions analyzed.

Few cases of human EMCV disease have been documented; however, in the older literature, virus isolation was reported from cerebrospinal fluid, blood, feces, and throat washings of patients (particularly children) with aseptic meningitis, poliomyelitis-like paralysis, encephalomyelitis, Guilláin-Barré syndrome, and fever of unknown origin (*10**–**12*). Human disease characterized by chills, fever, severe headache, stiff neck, pleocytosis, delirium, delusions, vomiting, photophobia, and fever also has been reported (*10*). A novel cardiovirus, SAFV, isolated from the stool of an infant with fever of unknown origin, recently was reported (*15*). SAFV and SAFV-like viruses also have been detected in nasopharyngeal aspirates from children with respiratory illness (*16*) or gastroenteritis (*17**,**18*). In all of these cases, however, virus was isolated only from specimens obtained from nonsterile sites, making the patients’ symptoms impossible to associate unequivocally with the cardiovirus infection. In other human picornavirus infections, virus can sometimes be isolated from whole blood, serum, or plasma, but viremia is usually of short duration and relatively low titer (*33*). For enterovirus infections, for example, blood is not considered a reliable source of virus, except in very young children (*34*). In infants, prolonged enterovirus viremia may lead to multiple organ involvement and more serious disease. In the 2 EMCV-infected patients described here, virus was isolated from serum collected 3 and 7 days after onset of symptoms, respectively, suggesting that viremia level was high and of long duration. Together, virus isolation from serum and, for 1 case-patient, documentation of >4-fold rise in antibody titer, provide conclusive evidence for causality.

Because few clinical or public health laboratories are capable of identifying cardiovirus infection, the effect on human health is unknown. The original detection and identification of SAFV used a combination of traditional virus isolation in cell culture and creation and characterization of a cDNA library by DNase sequence–independent single-primer amplification (*15**,**16*). A panviral DNA microarray also has been used to detect SAFV and SAFV-like human cardioviruses (*18*). Such laborious methods are not practical for routine diagnostic testing. Direct detection of human cardioviruses by RT-PCR, as described here, or by SAFV-specific RT-PCR (*17*), can be adapted for use in the clinical laboratory, and sequencing of PCR products can be used for confirmation and more detailed molecular epidemiologic analysis. On the basis of an analysis of SAFV sequences, the PCR assays described here should efficiently amplify and detect SAFV and all other known cardioviruses (data not shown), providing a valuable tool to detect cardioviruses in human specimens. However, before routine testing of human specimens can be justified, studies are needed to assess the prevalence of cardiovirus infection in human populations, to associate specific disease syndromes with cardiovirus infection, and to identify reservoir species involved in zoonotic transmission. These studies will help estimate the impact of disease caused by cardiovirus infection and identify prevention and intervention strategies.
